# A qualitative exploration of older people’s experience of discharge from mental health inpatient settings

**DOI:** 10.1080/17482631.2025.2581398

**Published:** 2025-11-12

**Authors:** Georgia Smith, Blair Hanlon, David J. Grinter

**Affiliations:** a School of Health and Wellbeing, University of Glasgow, Glasgow, UK; b NHS Greater Glasgow and Clyde, Glasgow, UK

**Keywords:** Patient experience, older adult mental health, inpatient, discharge planning, reflexive thematic analysis (RTA)

## Abstract

**Background:**

Recently here has been interest in the patient’s voice within community mental health settings as a catalyst of service development. There remains a lack of literature documenting the lived experience of inpatient mental health care in Older People’s Mental Health (OPMH) inpatient settings. There is a drive within the National Health Service (NHS) to transition care to community settings and improve the discharge process.

**Aims:**

To gain insight into older people’s experiences of being discharged from OPMH inpatient settings.

**Methods:**

Semi-structured interviews were conducted with seven people discharged from OPMH inpatient wards. They were asked to share their experiences of preparing for discharge, the support during this process, the transition to home, and what should be learned from their experiences. The interviews were analysed using reflexive thematic analysis.

**Results:**

Six main themes were identified. 1) Discharge as a gradual process, 2) Feeling involved in discharge planning; feeling empowered, 3) Communication; contrast between positive and negative experiences, 4) Social support; a sense that there was a causal link between support and successful outcome, 5) Importance of nurse support; the benefits of having support, 6) The importance of readiness for discharge; the relationship between how the patient felt before and after discharge.

**Conclusions:**

This is one of the first studies to explore older people’s experiences of being discharged from mental health hospitals. It provides insight of the factors that patients believe are important for a positive experience of discharge. Suggestions for service improvements and recommendations on how patients are supported appropriately in the process are discussed.

## Introduction

In line with government policy, reducing the length of time that patients stay in the hospital has been a key priority for NHS Scotland for many years. The Scottish Government (2021) has recently introduced two new programmes—“Interface Care” and “Discharge without Delay”—which aim to explore alternatives to inpatient care and improve the discharge process for those who require admission. Hospitalization has been linked to disrupted sleep, poor nourishment, changes in medication, mentally challenging situations, and deconditioning associated with inactivity and bed rest (Krumholz, 2013). Currently, emergency admissions for the elderly population are of particular focus. Mental health problems in older people present with higher levels of complexity and associated health and social care costs, with patients aged 75 years and over experiencing longer hospital stays, a higher risk of healthcare–associated infections, delayed discharge, and institutional care outcomes (Public Health Scotland, 2020). The proportion of older adults in acute care hospitals is over 40%, and almost 40% are discharged to a higher level of care than they have on admission (Royal College of Psychiatrists, 2019). Strategies aimed at reducing the likelihood of hospital admissions have received considerable research attention, including innovations for more collaborative or user–focused approaches (Wright et al., 2015). The transition period from inpatient mental health to the community has been found to be the greatest risk for adverse outcomes, including relapse, rehospitalisation, and suicide (Chung et al., 2017). Osborn et al. (2021) found that within their UK sample, 21.4% of individuals discharged from inpatient mental health care settings were readmitted within 6 months, and older age was identified as a statistically significant risk factor for readmission. Loneliness, limited social networks, and difficulties in attending appointments due to lack of transport (Beebe, 2010) have been cited as reasons for the difficulty of the post–discharge period. Older adults are at an increased risk for all stressors. Mental healthcare has changed globally with greater emphasis placed on community care by health care providers (Cohen & Minas, 2017) and as a result there will be greater need for good quality discharge planning. Whilst there is a trend towards care outside of institutional settings, it is recognized that in some parts of the world the lack of resources has limited that modernization of mental healthcare, despite some successes in development being achieved. This is the case in some parts of sub–Saharan Africa (Atewologun et al., 2025) and disparities in care models across Europe (Vandoni et al., 2024).

Quantitative methods have typically been used in psychiatric discharge research and have covered a range of topics, including suicide rates, hospital readmission, and effectiveness of discharge interventions (Meehan et al., 2006; Puschner et al., 2011). However, relatively few studies have sought to consider and explore patients’ experiences of discharge and transition from inpatient to community, and the research that has been done has been with those under the age of 65 (e.g., Jesus et al., 2024; Krupchanka et al., 2017). Evidence has demonstrated that patient experience feedback can assist services to better meet needs, result in more efficient and effective use of services, and positively affect length of hospital stay (Doyle et al., 2013). Redding et al. (2017) used Interpretive Phenomenological Analysis (IPA) to understand the lived experiences of adults (mean age of 46 years) who were recently discharged from mental health inpatient settings. They highlighted many common themes between the participants’ accounts, including difficulties in adapting to the community and the importance of support on discharge. Participants felt that linked care between inpatients and the community would ease their anxiety. The Mental Welfare Commission for Scotland’s (2019) paper on peoples’ lived experiences of psychiatric hospitals also highlighted people’s expressions of the need for access to adequate care and support on discharge. Transitions in care involve a multitude of health and social care professionals working within and across different organizational boundaries (Waring et al., 2014). The King’s Fund (2015) highlighted the pressures that hospitals face when discharging and transferring patient care, which has led to inappropriate assessments and readmissions and a lack of communication between the health and social care sectors.

The limited research available on patient experience within older people’s mental health care has thus far focused on community services. Comparisons between the patient experience of older people (≥50 years old) with and without serious mental illness in the United States indicated that older adults with mental health disorders reported significantly worse provider communication as well as the greatest barriers to shared decision–making and accessing services (Fortuna et al., 2017). Communication problems have also been reported as a key care experience for older adults accessing community mental health services in the United Kingdom (Wilberforce et al., 2018), together with personal qualities and relationships, feeling powerless, in–and–out care, bureaucracy, a focus on life, mental health, and continuity of care. To our knowledge, there have been no studies exploring older people’s experience of the discharge and transition process from mental health inpatient settings to the community, despite the associated risks.

## AIMS and research questions

The current study had three main aims:

- To explore older people’s experiences of the discharge process and the transition from mental health inpatients to community settings.

Specific research questions were as follows:

1. How do older adults characterize their experience of discharge from a mental health inpatient hospital?
2. What aspects of the process do they describe as beneficial or unhelpful?

## Methods

### Ethical approvals and considerations

Ethical approval was obtained from the West of Scotland Research Ethics Service (reference: 24/WS/0003, IRAS: 330114) and management approval obtained from NHS GG&C Research and Innovation. All research involving the recruitment of participants from health services in the UK requires ethical approval from an NHS Research Ethics Committee (NHS Health Research Authority, 2023). Written informed consent was obtained from all participants, and the recording and electronic storage of confidential patient information adhered to the Data Protection Act 2018 (Data Protection Act, 2018) and the General Data Protection Regulation (GDPR; Council regulation (EU), 2016/679, 2016). This work was part of the first author’s Doctorate in Clinical Psychology thesis at The University of Glasgow (Smith, 2024).

### Participants

Participants invited for one–to–one interviews were patients who had been discharged from one the older people’s functional mental health inpatient wards in a Scottish Health Board. These wards provide assessment and treatment for older people with acute “functional” mental illnesses. The term “functional” mental illness applies to mental health disorders other than dementia, such as mood disorders (Hatfield et al., 2011). Patients invited to participate must have been discharged from inpatient to community care within the 12 months preceding the interview; however, those discharged within one month were excluded to ensure no disruption to their transition process. Participants were aged 65 years or older at the time of discharge, recruited through the Older People Community Mental Health Teams (OPCMHT), and must have been deemed by care coordinators as psychologically well enough to participate. Patients diagnosed with dementia, delirium, florid psychosis, problematic substance use, or a lack of capacity to consent (as determined by their care team) were excluded. Guest et al. (2020) findings from a bootstrapping analysis of thematically coded qualitative datasets indicate that six to seven interviews capture the majority of themes in a homogenous sample. Therefore, we aimed to recruit–6−8 participants, to account for potential participant withdrawal from the study.

### Procedure

The eligibility criteria and Patient Information Sheet (PIS) were circulated to OPCMHT clinicians via email, and clinicians were asked to share the PIS with potential participants. Participants were asked to “opt–in” to the study by i) contacting the researcher or ii) providing verbal consent for clinicians to share their contact details with the researcher for them to make initial contact. The researcher contacted all participants via telephone, providing opportunities for questions regarding participation. The participants were invited to choose the date, time, and mode of the interview. Modes of interviews offered were face–to–face in a clinic meeting room at a health or resource center of their choice or videocall using Microsoft Teams. Participants were provided with the opportunity to claim their travel expenses to and from the interview location. Participants were also invited to bring a loved one or carer to the interview to provide support if required.

A semi–structured interview schedule was developed in consultation with an expert with experience employed by the Mental Health Network. They provided guidance on the use of appropriate language/jargon, as well as insight into common hospital experiences and/or journeys for older people, to ensure that the questions allowed participants an opportunity to discuss this. Utilizing this interview schedule, participants were asked to discuss their experience of preparing for discharge, the care and support they did or did not receive during this process, the transition from hospital to community, and what they think services could and should learn from their experiences of leaving the hospital. Participants were informed that it was their experiences that the researchers were seeking to understand; therefore, although the interview was semi–structured, the content could be guided by them, including decisions on what they did not wish to discuss.

All interviews were conducted by one researcher and audio recorded and transcribed verbatim by the same researcher, with identifying information removed, de–specified, or pseudo–anonymised. Participants were asked to complete written consent forms, were provided with a copy, and were advised to contact the researcher if they had any concerns regarding the content following the interview. The interviewer spent a brief period before each interview, building rapport with participants, as well as providing a chance to reflect on their interview once it had been completed. Participants were also provided with a post–interview support resource, which provided guidance and contact numbers for accessing support, as well as the complaints procedure and contact details for the Health Board.

### Analysis

Interview data were analyzed using Braun and Clarke (2006, 2022) six–phase process of reflexive thematic analysis: familiarization with the dataset; coding; generating initial themes; developing and reviewing themes; refining, defining, and naming themes; and writing up. This approach has been widely used and accepted as robust across a wide range of disciplines (Braun & Clarke, 2013). Braun and Clarke (2006) 15-point checklist for reflective thematic analysis was utilized to ensure the fidelity of the approach. Given that there is little to no prior research in this area, an inductive, semantic, and data–driven approach was adopted while also recognizing the role of the researcher in the co–creation of themes (Braun & Clarke, 2013). The analysis was conducted within an experiential, realist, and essentialist qualitative framework, aiming to capture and explore patient and staff perspectives, understanding, and reality.

### Researcher characteristics and reflexivity

In reflexive TA, the researcher plays an active role in the production of knowledge, where codes are understood as interpretations of patterns of meaning across a dataset (Braun & Clarke, 2019). Reflexivity critically examines how one’s own experience, knowledge, and social positioning influence the research process. In the current study, the researcher’s positionality is shaped by their identity as a young, white British female with a middle–class background, holding a postgraduate education, and working as a Trainee Clinical Psychologist. This research was conducted as part of the researcher’s Doctorate in Clinical Psychology.

The researcher’s clinical experience predominantly lies in Older People’s Mental Health (OPMH) services, which provide a foundational understanding of the specific needs of this population. This familiarity may have influenced how they interpreted the participants’ experiences of discharge, potentially leading to a focus on narratives that align with clinical experiences or established psychological theories. The primary researcher had not worked specifically within OPMH inpatient settings; thus, “outsider” status provided a degree of objectivity and an opportunity to approach data collection and analysis with curiosity. Additionally, this researcher identified as an “outsider” in terms of not being an older adult who has experienced psychiatric hospital admission. However, they have personal experience as close family members of someone who has undergone this experience, which may have shaped their emotional engagement with the research topic and introduced biases, such as over–identifying with certain narratives or emphasizing emotional aspects over other dimensions of the participants’ experiences.

To mitigate these influences, minimize bias, and ensure that the research remained grounded in participants’ perspectives, the researcher employed several strategies, including maintaining a reflective journal to regularly document and critically reflect on their biases, assumptions, and emotional responses. This was further complemented by regular research and peer supervision, as well as by two researchers independently coding the three interview transcripts. A commitment to reflexivity guided the analysis, with particular attention paid to avoiding over–reliance on preconceived ideas and remaining open to the full range of participant experiences.

## Results

### Participant demographics

Seven patients were interviewed for this study. The researcher was made aware of the 17 eligible participants who had been contacted by their clinician regarding the study. Of these, 10 expressed interest in participating and consented to further contact with the researcher. Of these, two declined participation; thus, eight opted to participate. Due to unforeseen circumstances, one interview did not take place and the participant opted out of the study. Seven participants were interviewed for this study. The mean age of the participants was 71 years old. The length of admission ranged from 3 to 6 months. All participants were discharged to their own home. The participant characteristics are shown in Table I. All participants were White Scottish or British in ethnicity. All participants opted for face–to–face interviews in the clinic space of their choice. Two participants opted to have a family member during the interview.

**Table I.** Participant characteristics.

Participant pseudonym	Age	Length of admission	Time since discharge
Sam	75	8 weeks	4 months
Alice	71	3.5 months	5 weeks
Evelyn	79	5 months	5 months
Charlie	70	6 months	5 months
Betty	66	3.5 months	2 months
Jane	68	5 months	11 months
Rowan	69	4.5 months	6 months

### Interviews

**Figure 1.**
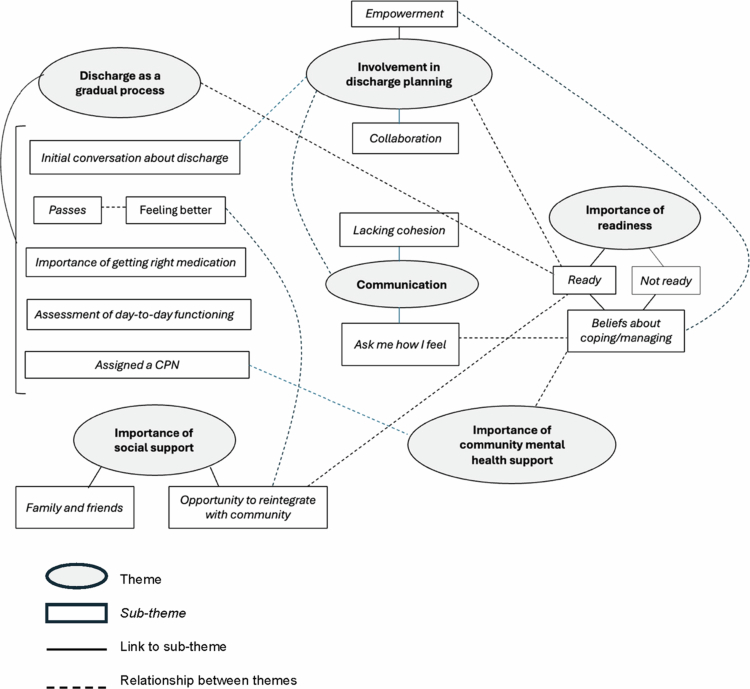
Thematic map.

Six main themes were identified: discharge as a gradual process, involvement in discharge planning, communication, social support, importance of community mental health support, and importance of readiness for discharge. A number of sub–themes were identified within each main theme, as presented in Figure 1. Thematic map.

#### Discharge as a gradual process

Participants described discharge as a gradual process, something that was worked on and built up, and the gradualness of this process was perceived positively by those who experienced it this way:

*“They worked it up… They don't just say* *‘Right, you'll go home tomorrow’, they gradually work at it, which is a good thing, I think.”* Alice

Participants spoke of their experiences of four main aspects to this gradual process.

***Subtheme: Initial conversation about discharge:*** Most participants were able to recall when discharge was first mentioned to them, although not all could recall at what point on their admission this conversation first took place. Three participants reported that discharge was first mentioned by a psychiatrist. One reported that their psychiatrist provided reassurance that they would be discharged soon and could recall their memory of what the psychiatrist said.

*“He says* *‘don’t you worry’ … ‘we’ll have you out, soon as. You won’t be in here for a long time. You just keep doing as you’re doing”*

Another participant reported that a conversation about discharge was initiated by their psychiatrist, asking them how they felt about going home. Three participants reported that they initiated conversations with their psychiatrist about discharge:

*“I mentioned it and at the time I did, I wasn't there very long.”* Betty

Some participants reported that the first time discharge was mentioned to them by a health professional was nurses, implying they would be going home soon:

*“It was just the nurses just used to say I could be getting home soon. You know, that was all, you know, they would sort of say to me ‘You'll be getting home soon’*” Evelyn

One participant reported that whilst nurses implied that they would be discharged soon, they were not told by anyone in “authority” (the participant clarified that senior nurses and doctors were the clinicians with authority). They reported that this was different from their experience of discharge in their previous admissions to the same ward.

*“Well actually, this last time, it was very strange because some of the nurses would say ‘Oh well, you’ll be getting out soon’. And nobody in authority ever told me.”* Rowan

***Subtheme: Passes and feeling better:*** All participants spoke of “passes” (temporary, unaccompanied leave from the ward which range from a few hours to several days) as part of that gradual process. Participants reported that the time spent on the pass increased throughout their admission, and the time and frequency at which this increased was reported as different for each participant:

*“They would take me out for a couple of hours, to the flat* [apartment]*. And then that built up—I ended up getting out overnight.”* Alice

*“But it was good what they done. You get your day passes and then your overnight stays, to build that relationship up with the new house.”* Jane

Passes were seen by the participants to help them prepare for discharge. One participant described the passes as follows:

*“…a way of introducing you to the outside world.”* Rowan

There was a sense of causal relationship between an increase in time spent on passes and feeling better. However, causality appeared to be experienced differently among participants. Some participants felt that the time out on and frequency of passes increased because they felt better. Others felt that this increase in time out on, and frequency of passes, led to them feeling better. One participant reported feeling that they would have benefited from a more “built up” approach to discharge:

*“So I think if I had the longer stretch of that … if I had the same as the other guys, maybe for about two months - I was getting out every second week or every week - it would have built me up more ready for coming home… I think I'd be getting out a lot easier than what I was.”* Charlie

They described witnessing other patients going out on passes for a longer period and at a higher frequency. Reflecting on this, they felt that going out on pass earlier into their admission, and for a longer time period, would have increased their feelings of readiness for discharge and made their discharge easier.

***Subtheme: The importance of getting the correct medication:*** Most participants spoke of the influence of medication on the way they felt, thought, and experienced their discharge. One participant felt that the effectiveness of medication positively affected their thinking style and made their experience in the hospital easier.

*“I'm finding that the tablets are working for me, you know. I think it made it a lot easier and the way I was feeling and my thoughts - my thoughts were always good. I just kept thinking positive all the time.”* Sam

Another participant spoke of the influence the dosage of their medication had:

*“But the second time they put me on a dosage and things just settled down.”* Alice

They spoke of being discharged from the hospital twice during the same care episode. They reported that their first discharge was unsuccessful, and that they were readmitted. They felt that when put on a different dosage of medication, the second time they were admitted, things settled down for them, and they associated this with remaining out of the hospital. One participant spoke of their psychiatrist cross–titrating their medications.

*“I think it was a combination … He wanted to try Venlafaxine, so he done what was called a cross titration. And whilst seeing the psychologist and him doing that - I think once that was in place and then psychology was enabling me to understand “Look its early days. You have to give it time” … So it did start to fall into place gradually.”* Betty

They spoke of the impact of this, alongside input from a psychologist, to enable an understanding of the influence of time on the effectiveness of medication. It appeared that they felt a combination of medication and psychological input, which led to things gradually improving and leading to their discharge.

***Subtheme: Assessment of day–to–day functioning***

*“So part of the discharge was that the occupational therapist, she would come and watch me frying. And it was good because I went home, I done everything that I was supposed to.”* Sam

Participants talked positively about functional occupational therapy assessments as part of their discharge process. They spoke of being asked to lunch while the occupational therapist observed and visited places in the local community. The participants recognized these functional assessments as part of the discharge process. One participant spoke of their hesitancy to return to their house and explained that they had started to discuss alternative living arrangements with their families. However, it appeared that the passes and visits with the OT increased their confidence in their ability to manage at home, which led to more certainty about their desire to return to their own house:

*“So, the OT actually took me home twice before I left. One of times she asked me to make lunch… And then another time she took me to a café. That was fine as well. And then I started to feel quite good … The fact that I'd made lunch and then I did it again … That kind of made-up my mind that I wanted to go home.”* Evelyn

***Subtheme: Assigned a community psychiatric nurse (CPN):*** All the participants spoke of being assigned a CPN upon or after discharge. This appeared to be a key part of the discharge process of the participants. Knowing that support would continue from the hospital to the community appeared to contribute to this participant’s confidence in how things would be discharged.

*“Because seeing the help I was getting, I knew I would still be getting the help from the community team and that, when I was out… The support that I had was great. You just knew that things were going to be ok.”* Jane

There was value placed on having continuity of care and assurance that they would not be left without support. This gave them confidence in both the system and their ability to cope with the transition. Some participants reported that their CPN was attended within the week after their discharge, and some reported that they experienced a delay in being assigned a CPN. When participants experienced a delay being assigned a CPN, they spoke of the reassurance and plan provided by the OPCMHT to contact the service if they required support. This subtheme is intrinsically linked to the main theme, “Importance of community mental health support.” Participants reported on the type of support received, and the impact this had, can be found in the description of this theme.

#### Involvement in discharge planning

Several participants reported that they felt involved in planning and decision–making regarding their discharge. Sub–themes of “empowerment” and “collaboration” were also identified due to participants recalling what their psychiatrist said, and how they said it, during conversations about discharge. Collaborative work and a sense of empowerment appeared to coincide together for participants. Generally, participants reported feeling listened to the following:

*“He listened to you, what decision you wanted rather than him dictating”* Alice

They also acknowledged that they were provided with choice and empowered to make their own decisions and trusted by clinicians in knowing how they felt about discharge.

*“’… it must be you that decides’, he says. ‘I’m here - to make suggestions to you. And if you feel that that's going too fast for you, we'll just take a step back and slow down.’”* Sam

*“He said ‘You’re the person that’ll know. You’ll know yourself.’”* Sam

Some participants stressed the importance of the discharge process as collaborative in nature, and did not feel that it was something in which they had no agency. There was a sense that collaboration resulted in the participants trusting the clinical team.

*“I think we worked in partnership and I had to be honest with them as much as they were honest with me.”* Betty

*“’We’ll try this’, in a lovely, relaxed—great feeling. I trusted him.”* Sam

One participant reported that they did not feel involved in discharge planning, and highlighted the lack of communication from their care teams as a problem during their admission. They thought that this was the biggest contributor to their lack of involvement in their own care.

#### Communication

Most participants spoke about the line of communication they experienced with the hospital staff regarding their discharge. This tended to be reflective of their experience of communication throughout their time in the hospital, and not just in relation to discharge.

***Subtheme: Lacking cohesion:*** For some participants, it appeared that they experienced communication among hospital staff, patients, and family members as they lacked cohesion. Although participants felt involved in decisions about their discharge, they did not always feel that they or other relevant people were informed of the plans for their passes or discharge. This appears to lead to miscommunication and feelings of confusion.

*“Sometimes I used to think what is going on here? How do they (nurses) know? And he's (psychiatrist) telling me something - you know, I just - a bit mixed up… It didn’t seem to gel together… Maybe a wee bit lack of communication.”* Alice

One participant spoke of what appeared to be a miscommunication with a nurse regarding when they were to return from a pass:

*“I went home on the Thursday and I came back on the Friday, and he went ‘What are you back for? You’re supposed to be - you could have stayed out till Monday’. But nobody told me. And I found out that a bit strange.”* Alice

Another participant spoke about the lack of communication between hospital staff and their family members, despite requesting that their family be informed:

*“And I said “would you make sure that my daughter knows how I am” … None of the nurses had got in contact with my daughter… The communication wasn’t good towards—for my daughter.”* Sam

When discussing their experiences of a lack of communication, this participant referenced a poster of the “Triangle of Care” which was up in the ward and stated:

*“That is not happening.”* Sam

The Triangle of Care is based on the core principle that carers, people who use services, and professionals should work in equal partnerships to promote safety, support recovery, and sustain wellbeing (Carers Trust Scotland, 2019). It appeared that for this participant, the lack of communication affected the way in which all three parties could work in equal partnerships to effectively support them.

One participant spoke about not knowing who to ask about passes, indicating that they were not informed of the appropriate line of communication regarding this:

*“I wondered how they always got out all the time. I know they had different doctors. And I didn't know who to ask.”* Charlie

***Subtheme: Ask me how I feel:*** This subtheme was identified because some participants felt that they would have benefitted from hospital staff providing an opportunity for them to communicate their feelings about readiness for discharge. One participant reflected on their experience and stated that if they were asked about how they felt about being discharged, they would have communicated their belief that they did not feel ready and would benefit from remaining in the hospital:

*“But I think if people ask me questions at the beginning when I got out, I could have said a lot more. You know, if somebody said to me—it was in my mind to say to them, but silly I didn't - … ‘How do you feel getting out now?’ … I think I would have said to them ‘I think I should be in there for another month or two. I'm not really ready. I'm not ready to face the world or face people.’”* Charlie

It appeared that the participants were aware, at the time, of how they felt about being discharged but would have benefitted from someone initiating and facilitating a space for them to voice their concerns.

Another participant talked about the benefits of being encouraged to think about life after discharge in their communication with their psychiatrist. This communication appeared to lead them to realize that they were not ready for discharge:

*“But he asked me questions like ‘Do you feel ready? How would you manage? How would you do your babysitting of your grandchildren?’. He enabled me to think about life and I’d think well, I'm not ready.”* Betty

The same participant appeared to experience effective communication around discharge planning between their health professionals as effective*:*

*“And obviously the psychologist was able to feed into that. she was able to speak to him and share information.”* Betty

#### Social support

The participants talked about the type and impact of the support they received from their family, friends, neighbors, and community groups throughout the discharge process. There appears to be a causal link between social support and a sense of reintegration into the community.

One participant was able to express this to their daughter, who was present during the interview:

*“Because I couldn’t have done that all myself without you. You've done all that. The talking and the tablets.”*

*“I probably would have been in there a lot longer, because I wouldn’t have had anybody to really sort of stick up for me.”* Sam

They spoke of their daughters, supporting them in resolving complications by accessing their medications on discharge. This participant felt that they would not have been able to resolve issues if they had not been for their daughter. They also felt that they would have remained in the hospital for a long time without advocacy from their family.

Another participant spoke about the inclusion of family and friends in their safety plan for extended passes from the ward.

*“So if I get a longer spell and have a safe plan, have the right phone numbers, the right family members, had some close friends. I would know what to do.”* Betty

When speaking about what they felt made their discharge successful, they spoke about the importance of:

*“Knowing that I had family, friends and there was a kind of plan from them about how they were gonna help.”* Jane

Some participants spoke about the support they received from their neighbors. They reported feelings of apprehension and anxiety about seeing their neighbors prior to discharge. This appeared to be tied to a sense of shame and fear of judgment about having been in a psychiatric hospital.

“*I didn't want them to know where I've been - even though they say there’s not, there is still a stigma being in a hospital like that.”* Alice

However, they went on to speak of the response they received from their neighbors upon returning home and how this disproved their initial fears:

*“In fact one of my neighbors came to the door on Friday night and she said ‘I was really worried about you’… And I appreciated her coming… so, people care.”* Alice

Another participant felt that they needed to move their house because their neighbors had witnessed the incident that led to their hospital admission:

*“And my biggest barrier for going home was - my neighbors came to my rescue that day … So I was really scared about that. I thought I needed to move; I cannot go back there.”* Betty

They explained that their psychologist helped them create a plan to face and challenge this fear by visiting their neighbors during an extended pass from the ward:

*“That's where psychology really helped me. We had a plan to go and see them before I was discharged, when I was on the more extended pass.”*

They reported that doing so helped to change their whole perception of going home:

*“And then the neighbor around the corner … She was so delighted to see me and so that changed my whole perception of home.”* Betty

Participants also spoke of the support they received from friends once they returned home. One participant spoke positively about the practical support offered by friends from the religious group they attended:

*“As soon as I got home, it was one of them says “I'll come and pick you up to take you to the meeting. I'll come and do this and do that. And that's all taken care of. You know, you don't even need to ask, they just offer. Which is very good.”* Evelyn

Another participant spoke positively about the support of a social group they attended, which appeared to be centered around mental health and wellbeing:

*“I go to a social group and it quoted as ‘It's OK to not be OK’. So that that has been a very good support.”* Rowan

#### Importance of community–based mental health support

When asked about the support received after discharge from the hospital, all participants spoke of the support they received from their older people community mental health service (OPCMHT), particularly their CPN.

Some participants reflected on feelings of hesitancy about being assigned a CPN upon discharge, which appeared to be related to their beliefs that they did not require further mental health input:

*“I kept saying ‘I don't really need a nurse coming out’. But as I said, it was a good thing because it gives you a wee bit sort of reassurance that there's somebody there.”* Sam

The importance of knowing their CPN at the other end of the phone was a shared experience for most of the participants. It appeared to provide them with reassurance and confidence that they would be sufficiently supported should they require it.

*“I know she's at the other end of a phone if anything was wrong.”* Alice

All participants felt that they were sufficiently supported by their CPN post–discharge and spoke of the range of ways in which their CPN provided support. Facilitating an opportunity for the participant to talk and actively listen was a form of CPN support experienced and valued by a number of participants:

*“She comes and she lets me talk. And she listens to me.”* Sam

*“It's just the way she talks to me. You know, as if she really cares. I know she does. I can tell.”* Evelyn

*“She listens. That's the main thing. And she can always be very positive about you.”* Rowan

Participants also described what appeared to be a strength–and goal–focused approach from their CPN, and this was perceived positively:

*“When they came they would speak about ‘What have you done? Let's concentrate on what you have done and how well you've done it and what would you like to do.’”* Jane

#### Importance of readiness for discharge

This final theme represents what appeared to be the relationship between participants’ views on their readiness for discharge and how they felt after being discharged.

***Subtheme: Beliefs about being able to cope/manage:*** Most participants reported a sense of worry before and after discharge. This was commonly related to their beliefs about being able to manage and cope with everyday life, such as engaging in activities of daily living, as well as related to a fear of negative evaluation from others about having been admitted to a psychiatric hospital, as discussed previously.

Some participants reported feeling ready for discharge. They spoke of feelings of happiness and excitement about returning home and of their initial fears and beliefs being disproved:

*“But when I saw the first friend, I found that really hard, but it went fine. My expectation of that wasn't how it turned out.”* Betty

For these participants, there also appeared to be a sense of wanting to move away from mental health services:

*“I don’t think I need anybody else, you know? I feel as if I'm just getting on with my life now.”* Sam

*“I've had enough. I don't want it, if you know what I mean. I just want to live my life now.”* Alice

Some participants reported feeling that they were not ready for discharge and were discharged too early. These participants spoke of an increase in feelings of anxiety since being discharged as well as fears of becoming unwell again:

*“But when I got out, at the beginning, I was saying to myself, I wish I stayed in for a bit longer. I don't think I'm ready. I felt as if I wished I was back in hospital after about 3−4 months… I felt I was a legal alien. I just wanted to hide in the house.”* Charlie

The relationship between feelings of readiness and post–discharge is represented well by one participant’s experience of being discharged twice. When speaking about the first time they were discharged when they felt they were not ready, they reported that they had stopped eating and felt a sense of blackness:

*“I wasn't ready the first time. And I felt they should have maybe known that… Because I remember getting home and it was on the Monday, I just went - it was just like a blackness… And I stopped eating and I just went right downhill. You know, I couldn’t cope.”* Evelyn

This contrasted with the way they spoke about how they felt the second time they were discharged when they felt ready.

*“I felt so much calmer than I did the first time… I just felt this is, this is where I should be.”* Evelyn

## Discussion

This study aimed to gain insight into older people’s experiences of discharge and transition by asking those discharged from the care of NHS Greater Glasgow and Clyde’s functional mental health wards to the care of older people community mental health services (OPCMHT). Through reflexive thematic analysis (RTA), we identified six common themes: discharge as a gradual process, involvement in discharge planning, communication, the importance of social support, the importance of community mental health support, and the importance of readiness for discharge. Several themes and subthemes were intrinsically linked.

Consistent with Redding et al. (2017) findings, discharge was perceived as a gradual process involving preparatory measures throughout admission. Disparities in participants’ descriptions of the discharge process indicate inconsistencies in discharge planning, which affects how patients feel pre–and post–discharge. Participants who reported feeling ready to be discharged shared their perception of discharge as a gradual process and reported active involvement in discharge planning.

These findings suggest that how discharge is initially mentioned and subsequently discussed with patients affects their perception of involvement in discharge planning. Being empowered by clinicians to make their own decisions and being trusted by clinicians in knowing how they feel about discharge contributed to participants’ feelings involved in discharge planning, as well as experiencing discharge planning as a collaborative process with their clinicians. These findings align with Rotter’s (1954) theory of locus of control, in which individuals believe they can influence their health outcomes through personal agency. This sense of involvement and collaboration with clinicians in discharge planning reflects a strong internal locus of control. An internal locus of control, where participants felt they had control over their recovery and outcomes, appeared to be linked to higher perceptions of coping ability and readiness for discharge. Conversely, a lack of involvement appeared to reinforce an external locus of control, undermining the participants’ confidence in their ability to cope. This finding contributes to the findings of numerous studies that an empowering approach in mental healthcare has been linked to the process of psychiatric recovery (Horsfall et al., 2017; Leamy et al., 2011; Wolstencroft et al., 2018) and highlighting the importance of empowerment and collaborative practices in psychiatric discharge planning for older people.

Passes were a key factor identified in the discharge process, and the increase in time–out on and frequency of passes appeared to be intrinsically linked to “feeling better.” These findings align with Bandura’s (1997b) self–efficacy theory, whereby passes serve as a mastery experience, providing opportunities to confront and manage fears about coping outside the hospital environment. By managing anxiety and becoming more comfortable with community reintegration, the participants appeared to experience an increase in self–efficacy, contributing to a stronger sense of readiness for discharge (Doctuer et al., 2022). This supports prior findings that self–efficacy plays a considerable role in psychiatric recovery (Barakat et al., 2021; Abraham et al., 2014) and demonstrates preliminary findings that passing during psychiatric hospital admission is a practical tool for building self–efficacy among older adults.

Findings also highlight the positive impact of social support and connectedness in fostering successful discharge and reintegration into communities, concordant with the principles of self–determination theory (Ryan & Deci, 2000). Social support, particularly from family members, fuls the basic psychological need for relatedness, increasing the sense of connectedness and support during hospital admission and discharge. Family members were also seen as advocates and key to the resolution of issues related to discharge, enhancing feelings of competence by helping participants navigate a complex system and regaining a sense of control over their lives.

Additionally, participants reported that they and their family members experienced a lack of cohesive communication from clinicians regarding their discharge plans and, more generally, throughout psychiatric hospital admissions. Recent literature has continued to highlight the key role of effective communication in ensuring satisfaction with the discharge process and engagement with post–discharge support and care (Nurjannah et al., 2014; Tahseen & Davies, 2023). Despite the promotion of the “Triangle of Care” (Carers Trust Scotland, 2019) within these settings, miscommunication and a lack of communication were frequently experienced by patients and their families. This lack of clear and consistent information can be seen as undermining the psychological need for autonomy, as participants and their families felt uninformed and unable to make empowered decisions regarding care and discharge planning. Taken together with patient experience reports of older people accessing community mental health services (Fortuna et al., 2017; Wilberforce et al., 2018), findings strongly indicate, in line with evidence (Gledhill et al., 2023; Tahseen & Davies, 2023) that improving issues with communication should be a key focus in OPMH clinical interventions.

Consistent with findings in prior research within the adult population (Redding et al., 2017), it is important to have someone to talk to or visit immediately post–discharge. In the current study, knowing support was available from the older people community mental health team and was vital to all participants both pre–and post–discharge. There was value placed on having continuity of care and assurance that they would not be left without support. Central to the support provided by CPN’s was having an opportunity to talk, being listened to, and being encouraged to take a strong and goal–focused approach to the transition from hospital to home.

There were several reasons why RTA was chosen as the data analysis method for this study. First, it is an easily accessible and theoretically flexible interpretative approach to qualitative data analysis. Second, in RTA, the researcher’s subjectivity is not considered a threat to the study findings or a negative source of bias (Braun & Clarke, 2022). Third, RTA is well suited to the exploration and understanding of patient experience because it allows for a flexible, in–depth, and nuanced exploration of complex and subjective phenomena.

Crucially, this study highlights how patient experience research offers patients the opportunity to identify areas of inefficiencies and improvements in service development from the perspective of an expert by experience. In sharing their experiences, the participants in this study offer valuable insight into how NHS Scotland, and other healthcare providers, can improve discharge planning and processes for older adults requiring psychiatric hospital admission.

### Limitations

Although this study had an appropriate sample size for a qualitative design, care must be taken in the transferability of the findings. This was a homogenous sample of white Scottish or British participants, all of whom had good family support and were able to travel to attend the interviews. Only patients who were deemed cognitively able and well enough to participate were provided information regarding the study. Patients with cognitive impairment or those who were not sufficiently well may have had different experiences. The longest admission was 6 months which, although longer than the national average for Scotland (4.5 months; Scottish Government, 2022), may have had an impact on the results as those patients with significantly longer admissions may have had different experiences. Additionally, this research was conducted within only one health board of NHS Scotland. The provision of care within NHS Scotland is changing and may vary across the country; therefore, it is acknowledged that experiences might differ.

### Clinical implications

Care teams should ensure that patients are supported in accessing time away from the ward, where safe to do so, and that they are involved in planning the time and frequency of these increases. Findings indicate that patients would also benefit from being encouraged to discuss their feelings about, and fears for discharge, and utilize passes as an opportunity to address and resolve concerns. Additional efforts should be made to ensure that patients without family or friends have opportunities to access both social support and advocacy during the discharge process. The findings also emphasize the importance of inpatient care teams adhering to the core principles set out in the Triangle of Care (Carers Trust Scotland, 2019) and ensuring that all relevant parties are informed of the discharge decisions and plans made. Globally, for those services that are transitioning from predominantly hospital–based care (Atewologun et al., 2025; Vandoni et al., 2024) there are opportunities to embedded patient–centered discharge planning into new practices, whilst in countries where deinstitutionalisation is at an advanced stage, a shift from current practices might be beneficial.

### Implications for future research

Understanding the clinician’s perspective of the discharge process is an important area for future research and would provide valuable insights into what procedures are in place to involve patients in planning and decision making regarding their discharge, how “readiness” for discharge is assessed or determined, and potential barriers to cohesive communication within OPMH settings. The association between patient involvement and readiness or the function of passes and readiness for discharge was not explored further in this study. Future research examining these concepts could provide useful information for reducing the length of hospital stay and the risk of readmission for those admitted to OPMH settings. Further research exploring the experiences and needs of older adults without family or social support could provide useful information on how the needs of this population can be appropriately met during psychiatric admission and discharge.

## Conclusion

To the best of our knowledge, this is one of the first studies to explore older adults’ experiences of being discharged from mental health hospitals. These findings support prior research findings that discharge from psychiatric hospitals is a gradual process and is dependent on a number of steps and factors. Factors that seemed particularly pertinent to older adults included passes, patient involvement in discharge planning, social support, and community mental health support. Patient feedback indicates the need to improve the cohesiveness of communication between patients, clinicians, and families/caregivers during discharge planning. Further research exploring clinicians’ perspectives on discharge would provide further insight into discharge planning processes and procedures, and identify barriers to patient involvement and cohesive communication.

## Acknowledgements

This work was part of the first author’s Doctorate in Clinical Psychology thesis at The University of Glasgow (Smith, G, 2024). The university publishes all of its students’ thesis work on its library website as a matter of routine. This work, although examined as part of the course requirements, has not been peer reviewed or published in scientific journals.

## Author contributions

**Georgia Smith** was involved in the design, analysis, and interpretation of the data. She was responsible for drafting the manuscript and revising the content for submission.

**Blair Hanlon** was involved in the conception and design of the study and provided feedback on the early drafts.

**David Grinter** was involved in the conception, design, analysis, and interpretation of the data. He provided commentary on the drafts of the paper and revised the content for submission.

## Data Availability

The data that support the findings of this study are available on request from the corresponding author, DG. The data are not publicly available due to restrictions [e.g., their containing information that could compromise the privacy of research participants].
